# Walking through doorways helps remembering, but not for long

**DOI:** 10.3758/s13421-025-01832-8

**Published:** 2026-01-09

**Authors:** Noah A. Crockett, Dani Parra, Abigail C. Doolen, Gabriel A. Radvansky

**Affiliations:** 1 University of Notre Dame, Notre Dame, IN USA; 2 Department of Psychology, University of Notre Dame, 390 Corbett Hall, Notre Dame, IN 46556 USA

**Keywords:** Memory, Mental models

## Abstract

The structure of events in which information has been learned can meaningfully impact memory, particularly for information that is encountered near an event boundary. Prior work has shown that separating a list of words into multiple events can improve later memory, suggesting that event structure could be leveraged for a benefit. That is, using event structure can help chunk a large set of information into more manageable units. Our aim was to replicate a prior finding that dividing a list of words to be learned in two rooms would improve later performance. Our results revealed that across two experiments, there was little evidence that memory was better when words were learned across two rooms rather than one. This did occur in one experiment, but only for words around the event boundary. However, this effect quickly dissipated after a short post-learning delay when a person moved through other rooms prior to testing.

The structure of events in which information has been learned can meaningfully impact memory. According to the Event Horizon Model (Radvansky, 2012; Radvansky & Zacks, 2011, 2014, 2017), the segregation of information into separate events can make retrieval easier when it serves to chunk knowledge. Moreover, information that occurs at event boundaries is prioritized because of increased attention, and is more likely to be remembered later (Swallow et al., 2009). The active segmentation and organization of information by extracting the structure of events is important for effective cognition (Richmond & Zacks, 2017). The current study further explored how memory for sets of information is influenced by whether it is learned as part of a single event or two events, and whether the information was learned at an event boundary.

According to theories of event cognition, we use event models when we process, comprehend, and remember information about events (e.g., Radvansky & Zacks, 2014). Event models are mental simulations of ongoing events, similar to the concept of situation models (van Dijk & Kintsch, 1983; Zwaan & Radvansky, 1998) and mental models (Johnson-Laird, 1983). When processing the dynamic world, we parse the flow of information at points when there are changes in the ongoing action (e.g., Newtson, 1973; Newtson et al., 1977; Swallow et al., 2009; Zacks et al., 2007; Zacks & Tversky, 2001). Changes in event components, such as a shift in spatial location, serve as event boundaries. As a result of this segmentation, information from each side of the boundary is stored in a different event model.

Some work suggests that memory is better when information is learned in multiple events rather than one. Specifically, when people parse the on-going information, this presents an opportunity to use the event structure to chunk and organize it (e.g., Zacks & Tversky, 2001). For example, in a study by Pettijohn et al. (2016), people learned word lists either within a single large room or in two rooms separated by a doorway. Across these experiments, memory was better when the information was distributed across multiple events than within a single event. This is broadly consistent with other research showing that people organize information into separate mental models (e.g., Zwaan, Langston, et al., 1995a, Zwaan, Magliano, et al., 1995b). The larger set of information could be chunked into separate traces, thereby facilitating retrieval (see also Sargent et al., 2013), which can be long-lasting (Flores et al., 2017). That said, no differences in adjusted ratio of clustering (ARC) scores^1^ (Roenker et al., 1971) were observed for the word list data, suggesting that there were no strong chunking influences on word list memory by the different events.

This is consistent with the more general finding that segregating information into different contexts can help memory. Some early work showed how moving from one place to another decreased retroactive interference (Bilodeau & Schlosberg, 1951; Greenspoon & Ranyard, 1957; Jensen et al., 1971; Smith et al., 1978; Strand, 1970). In these studies, people were given two lists of items, one in one room and another in either the same room or a different one. The studies found that there was less retroactive interference for the first set after a spatial shift relative to when there was no spatial shift. Moreover, Smith (1982, 1984; Smith & Rothkopf, 1984) showed that there was better memory for a list when people learned it in multiple locations.

Beyond the structured organization, other work in event cognition has suggested that when people parse ongoing information into separate events, they tend to better remember information around the event boundaries (e.g., Newtson, 1976; Swallow et al., 2009). This is because the change that occurs when there is a shift from one event to the next is a point of high attention. The state of the world has gone from one set of conditions to the next. Because there is more attention paid to information around the event boundary, this information is more likely to be remembered later.

The current study builds on one reported by Pettijohn et al. (2016). People were asked to learn two sets of 40 words. Each of these two lists was broken down into two sub-lists of 20 words. In a No Shift condition, people received the first 20 words at a location on one side of a long room, and the other 20 on the other side of the same room. However, in a Shift condition, people received the first 20 words in a shorter room and then walked through a doorway to a second room of the same size and were given the second set. Note that the distance travelled was the same in both of these conditions. Consistent with the idea that event segmentation can improve memory, there was better overall memory in the Shift than the No Shift condition. However, an analysis of ARC scores failed to show any differences in the chunking of the word lists in the two conditions. Moreover, although there was a trend in that direction, there was not better memory for the boundary items compared with the nonboundary items.

Thus, this study showed evidence that event segmentation improves memory for some aspects of event cognition (better overall memory), but not others (clustering and boundary item memory). It is not clear why the evidence is mixed. It may be because each condition was tested only once per person. Moreover, testing was given immediately after learning. Some aspects of memory emerge only after some time has passed (e.g., Roediger & Karpicke, 2006). As such, it might be better if some delays were imposed after the initial learning to allow for such changes to emerge. Additionally, this design could be improved by testing each participant multiple times in both the No Shift and Shift conditions.

To this end, the current study was a conceptual replication of the Pettijohn et al. (2016) study with three major differences. First, we used a virtual rather than a real environment. Virtual environments allow people to navigate on their personal computers. They enable a high degree of experimental control to reduce random variation while still allowing some ecological validity (Smith, 2019). Second, rather than a single trial for each participant to assess the influence of an event shift, there were multiple trials for each participant. Finally, for Experiment 2, we also explored the influence of the number of intervening rooms between learning and testing. Prior work has shown that memory for information grows weaker with more rooms (events) between where the item was placed and the current location it is in (Bower & Morrow, 1990; Bower & Rinck, 2001; Curiel & Radvansky, 2014; Haenggi et al., 1995; Millis & Cohen, 1994; Morrow et al., 1987, 1989, 1994; Rinck & Bower, 1995; Rinck et al., 1996, 1998; Stine-Morrow et al., 2002; Tamplin et al., 2013; Wilson et al., 1993; Zwaan et al., 1998). However, how this increased event distance influences the impact of event structure on memory has yet to be explored.

## Experiment 1

The aim of Experiment 1 was to assess the impact of event boundaries on word list memory as was done in the Pettijohn et al. (2016) study. However, we used a virtual rather than a real environment. We also assessed memory for boundary versus nonboundary words, as well as the degree to which the word lists were organized during recall based on list half.

### Method

#### Participants

Forty-one undergraduates (27 women, ages 18–22,* M* = 19.03, *SE* = 0.17) were recruited from the University of Notre Dame Department of Psychology participant pool in exchange for credit towards their psychology course. The sample size was determined based on Pettijohn et al.’s (2016) report, along with the fact that the current study had more trials per person (12) compared with the earlier study (2). All participants were 18 or older and had normal or corrected vision. This research was approved by the Institutional Review Board at the University of Notre Dame.

#### Materials

**Fig. 1:**
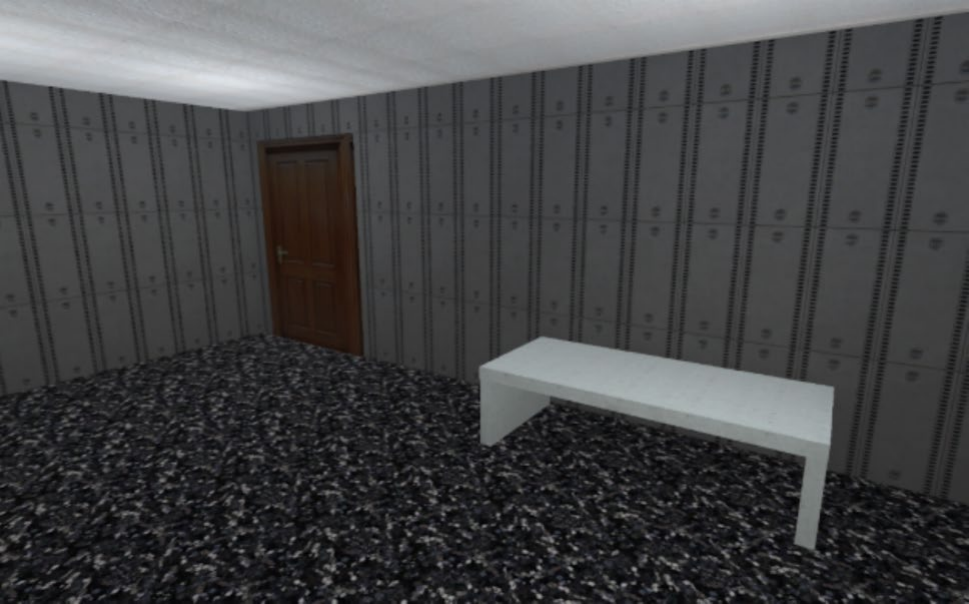
Screenshot of the virtual environment used in the study

Data was collected online using a virtual environment programmed with Unity® (www.unity3d.com). A screenshot of the virtual environment is shown in Fig. 1. The environments consisted of a series of rooms. These rooms were either large rooms with no wall or doorway between them or pairs of small rooms with a wall and doorway separating them. The two small rooms together equaled the size of a large single room. Within the rooms were tables that people would approach to see the learning materials. There were two tables in the large single rooms and only one table in each of the small rooms. The distance traveled between the two learning locations was kept constant. The only difference was whether there was a wall with a doorway between the two locations, which effectively divided the learning experience into two events. In addition to these rooms, there was also a small testing room with one table after each large single room, or after each small multiple-room pair. In the testing room, people approached the table and were then prompted to recall the words seen in the list. The virtual environment was displayed to the participants online using their own devices.

A set of twelve 20-word lists were generated. These words were 5 letters long and had scores of 20 to 103 on the Kučera and Francis (1967) word count norms, with an average of 45.1. These word lists were randomly generated for each participant. The list of the words used is provided in an online supplement.

#### Design and procedure

**Fig. 2:**
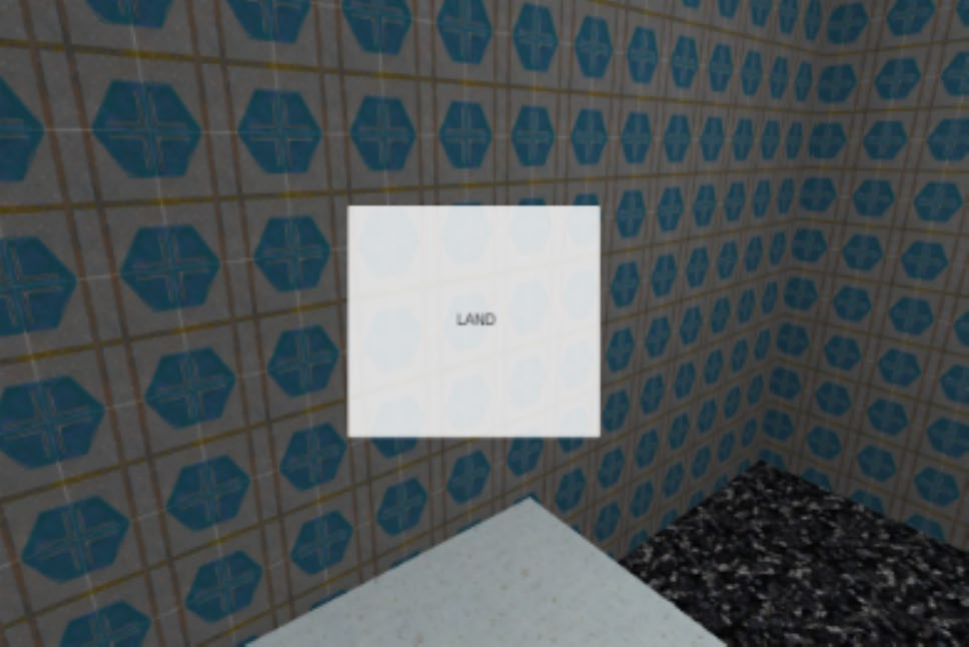
Screenshot of word list presentation within the virtual environment

All data were collected online with participants using their own computers. After providing informed consent and acquiring basic demographic information, people read the instructions and started the experiment. They initially navigated through three rooms to acclimate them to the virtual environment. Navigation was done using the *W*, *A*, *S*, and *D* keys on the computer keyboard, and motion was continuous. Mouse movement was used to change the point-of-view in the environment. After this, the learning phase began. When they approached a table in the learning rooms, a list of ten words appeared on the screen, one at a time, for one second each. An example of a screen shot is shown in Fig. 2. After this, people moved on to the second table where they were shown the second half of the 20-word list. If there was no change in location, then people walked across a large room to a second table. This is referred to as the *No Shift* condition. However, if there was a change from one room to the next, people walked through a doorway into a second room and approached the table there. This is referred to as the *Shift* condition. Half of the word lists learned involved the No Shift condition, and half the Shift condition.

After the second table, people walked through a doorway into another room. This was the testing room. Upon approaching a single table, people were prompted to recall as many of the 20 words as they could remember by typing them into a text box. They indicated that they were done with recall by pressing a button that said “Done.” This cycle of word lists and testing was repeated 12 times, six each in the No Shift and Shift conditions.

Note that having memory testing in a separate room from where the learning occurred is a departure from the Pettijohn et al. (2016) study. In that study, testing was always done in the second room that the learning occurred in. This introduced the possibility of an element of encoding specificity (Tulving & Thompson, 1973) into the procedure that has been bothersome to us for some time now. Having testing in a separate room removes this element. Given this, it should be noted that the terms “No Shift” and “Shift” refer to the presence or absence of an event shift within the set of information to be learned, while acknowledging the point that there is always a shift to the testing room, which is clearly a separate event from the list learning portion that came before it.

#### Analysis

Memory was assessed by measuring the proportion of words recalled. A score of 1 would indicate perfect recall. Moreover, we divided the words into Boundary and Nonboundary words. Boundary words were those in positions 10 and 11 that straddled the event boundary. This is where a shift from one location to the next occurred. Following Pettijohn et al. (2016), we also calculated adjusted ratio of cluster (ARC) scores (Roenker et al., 1971). For this measure, list halves were used as the categories. An ARC score of 1 would indicate perfect categorization and an ARC score of 0 would indicate random organization. Therefore, greater clustering by event would be evidenced by larger ARC scores. Analyses of variance (ANOVAs) were done in JASP (JASP Team, 2024) to measure the differences in accuracy between the single and multiple location conditions, as well as non-boundary and boundary words.

### Results and discussion

**Fig. 3:**
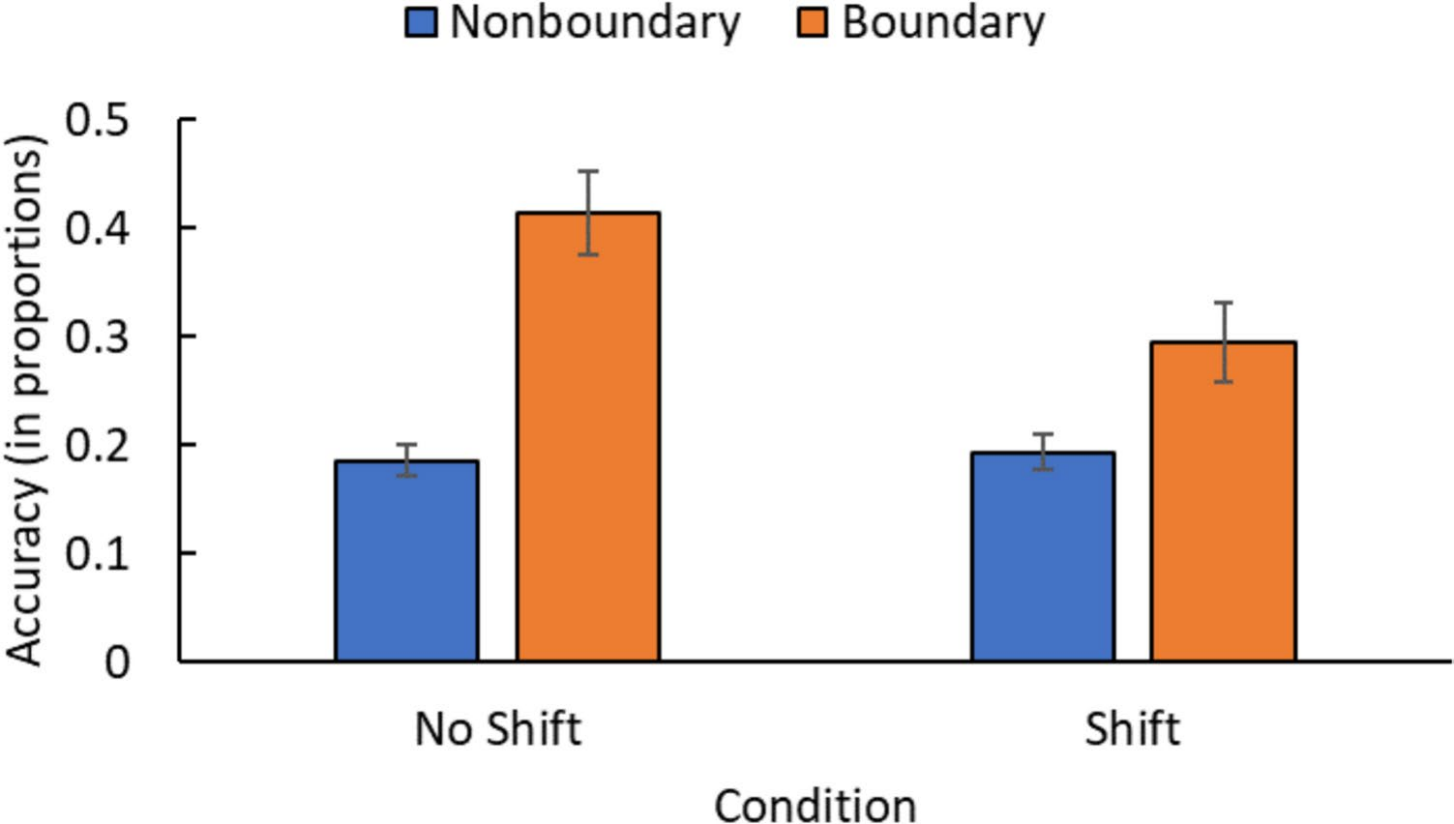
Accuracy scores for the No Shift and Shift conditions for Experiment 1 as a function of whether the words were Boundary or Nonboundary items

The mean recall accuracy rate for the memory test was .21 (*SE* = .02). The pattern of data is shown in Fig. 3. The accuracy data were submitted to a 2 (Event Shifts: No Shift vs. Shift) × 2 (Boundary: Nonboundary vs. Boundary) repeated-measures ANOVA. For this analysis, the main effect of Event Shifts was marginally significant, *F*(1, 40) = 3.40, *p* = .07, *η*_*p*_^*2*^ = .08, with people being slightly more accurate in the No Shift than the Shift condition. There was a main effect of Boundary, *F*(1, 40) = 46.71, *p* < .001, *η*_*p*_^*2*^ = .54, with memory being better for the Boundary than the Nonboundary words. Importantly, the interaction was significant, *F*(1, 40) = 5.29, *p* = .03, *η*_*p*_^*2*^ = .12.

This interaction indicates that for the Boundary items, there was an effect of Event Shifts, *F*(1, 40) = 4.69, *p* = .04, *η*_*p*_^*2*^ = .11, with people remembering less when there was an event shift compared with when there was not. This is the opposite of what was reported by Pettijohn et al. (2016). However, for the Nonboundary items, this difference was not present, *F*(1, 40) = 0.19, *p* = .66, *η*_*p*_^*2*^ = .01. Thus, we see some evidence that there was actually worse memory when information was segregated into multiple events, rather than just one.

An analysis of the ARC scores revealed no difference between conditions, *F*(1, 40) = 0.49, *p* = .49, *η*_*p*_^*2*^ = .01, with ARC scores being similar in the Shift (*M* = .23; *SE* = .02) and No Shift conditions (*M* = .22; *SE* = .02). Thus, there was no evidence of event structure affecting the chunking of information. Overall, the pattern of data for the memory test seems at odds with that of Pettijohn et al. (2016). There was no overall benefit of a Shift condition, although there was a trend in the opposite direction. Moreover, there was not more clustering. Also, while Pettijohn et al. found no difference between memory for Boundary and Nonboundary items, a difference was present here.

**Fig. 4:**
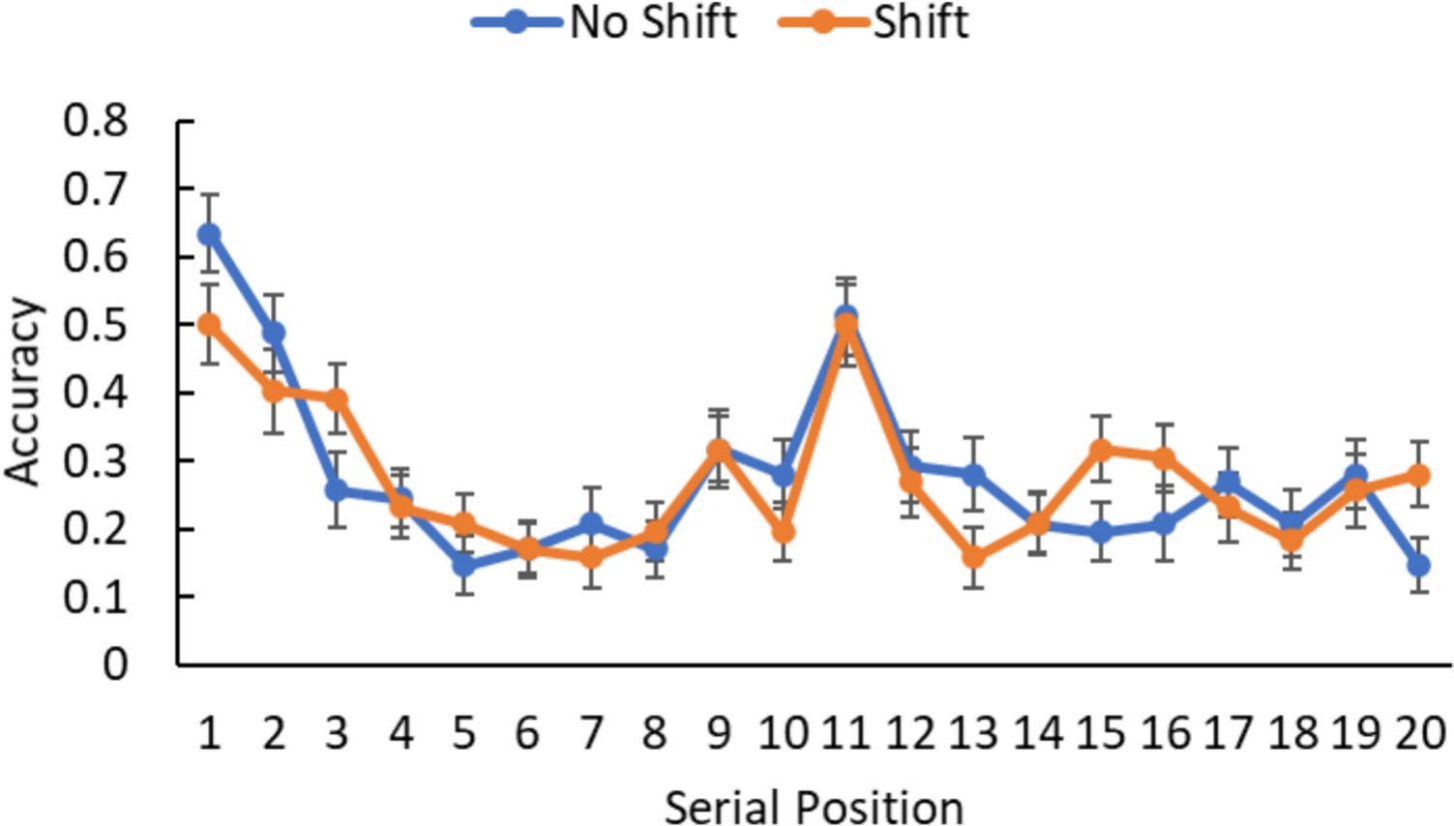
Serial position curves for the No Shift and Shift conditions of Experiment 1

As one final analysis, we consider serial position, and whether an event boundary might influence it. The classic serial position curve is a u-shaped function, with better memory for items early in a list (a primacy effect), as well as better memory for items at the end (a recency effect; e.g., Madigan & O’Hara, 1992; Murdock, 1962; Rundus, 1971). Although we used 20-word long lists, it was apparent that these were two sub-lists of ten. People either were given both halves in the same room, or were given one half in one room, and the other half in another. Thus, we can expect a double-U-shaped function with a recency effect at the end of the first list half, and/or a primacy effect for the first portion of the second list half. The important question is whether such an increase in memory in the middle would be accentuated by a shift from one room to another? The results of this assessment are shown in Fig. 4. As can be seen, while there is no recency effect for the end of the first list half, there is a primacy effect for the second list half. This suggests that the boundary effect reported earlier is primarily a primacy effect for the second list half. When the data were submitted to a 2 (Event Shifts: No Shift vs. Shift) × 20 (Serial Position) repeated-measures ANOVA, while there was a main effect of Serial Position, *F*(19, 760) = 10.68, *p* < .001, *η*_*p*_^*2*^ = .20, there was neither a main effect of Event Shift, *F*(1, 40) = 0.01, *p* = .92, *η*_*p*_^*2*^ < .001, or an interaction, *F*(19, 760) = 1.37, *p* = .13, *η*_*p*_^*2*^ = .03. Thus, the event shift did not alter the pattern of performance.

## Experiment 2

The aim of Experiment 2 was to further assess the impact of event boundaries on word list memory using a virtual environment with more observations than were present in Experiment 1, and with different levels of separation, in terms of intervening events between viewing and testing. The increase in the number of observations was done to leverage greater statistical power with the hope of observing an effect if one was indeed present, given that no such effect was seen in Experiment 1. The inclusion of the additional rooms between list learning and test was done to assess the stability of any patterns that we would observe. It is well-known that memory changes over time (Ebbinghaus, 1885). Rather than only testing immediately after learning, a delay was introduced in this way. Work in memory retrieval and longer delays has shown that sometimes differences between conditions can grow smaller (e.g., Staugaard & Berntsen, 2019), stay the same (e.g., Wixted & Ebbesen, 1991), or even grow larger (e.g., Roediger & Karpicke, 2006). Increasing the number of rooms between learning and test provided an opportunity to assess this without relying strictly on a simple-minded time delay. Event cognition research has shown that the number of intervening events can have an impact on memory retrieval, with greater distances between locations resulting in more difficult retrieval (e.g., Morrow et al., 1989). Moreover, it is the number of rooms that would have an impact on memory, not their size (Rinck et al., 1997).

### Method

#### Participants

A total of 240 undergraduates (151 women, ages 18–22,* M* = 19.23, *SE* = 0.07) were recruited from the University of Notre Dame Department of Psychology participant pool in exchange for credit towards their psychology course. Their ethnicities were 155 White, 34 Hispanic, 26 Asian, 13 Other, 9 Black, 2 preferred not to answer, and 1 Native American. There were 80 participants in each of the intervening rooms (0, 1, or 2) conditions. The increase in sample size was done to greatly increase the probability of detecting an effect, if it were present. All participants were 18 or older and had normal or corrected vision. This research was approved by the Institutional Review Board at the University of Notre Dame.

#### Materials and procedure

The words used in Experiment 1 were from the same pool as those in Experiment 2, and again used twelve 20-word lists. Like Experiment 1, the lists of words were learned in either a single room or across two rooms. However, the online, virtual environment was modified so that prior to the test room, there were 0, 1, or 2 intervening rooms. Thus, for 0 intervening rooms, the person walked from the room with the second list of words directly to the testing room. In comparison, for the 1 and 2 intervening room conditions, people walked through one or two rooms prior to entering the testing room. This was done to assess whether events between learning and testing would impact the pattern of results. The number of intervening rooms was manipulated between participants, thereby giving us three groups. Other than this, the procedure was identical to Experiment 1. The data were analyzed like Experiment 1, with the addition of a variable for the number of intervening rooms.

### Results

The presentation of results is divided into two major sections. First, we assess performance for people when there were no intervening rooms between the end of study and the memory test. This is done to assess the degree to which we were able to replicate Pettijohn et al.’s (2016) study. Following that, we assess how these patterns may change with increased separation between viewing and memory testing.

#### Immediate test

**Fig. 5:**
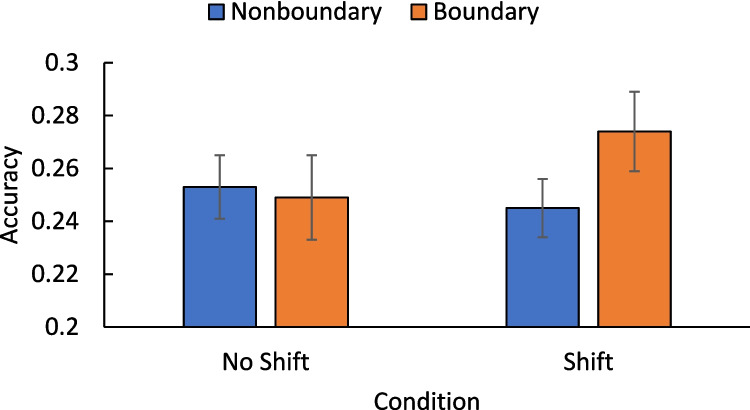
Accuracy scores for the No Shift and Shift conditions as a function of whether the words were Boundary or Nonboundary items

The mean recall accuracy rate for the immediate test was .25 (*SE* = .01). The pattern of data is shown in Fig. 5. The accuracy data were submitted to a 2 (Event Shifts: No Shift vs. Shift) × 2 (Boundary: Nonboundary vs. Boundary) repeated-measures ANOVA. This analysis revealed no main effects of Event Shifts, *F*(1, 79) = 2.29, *p* = .13, *η*_*p*_^*2*^ = .03, or Boundary, *F*(1, 79) = 2.31, *p* = .13, *η*_*p*_^*2*^ = .03, however the interaction was significant, *F*(1, 79) = 6.63, *p* = .01, *η*_*p*_^*2*^ = .08.

Breaking this interaction down, for the Boundary items, there was an effect of Event Shifts, *F*(1, 79) = 4.93, *p* = .03, *η*_*p*_^*2*^ = .06, with people remembering more when there was an event shift compared with when there was not. However, for the Nonboundary items, this difference was not present, *F*(1, 79) = 1.69, *p* = .20, *η*_*p*_^*2*^ = .02. Thus, we see some evidence that there was better memory when information was segregated into multiple events, rather than just one, and that this benefit was particularly evident for items that were encountered around the event. This finding is in contrast with the finding in Experiment 1 that showed worse memory for boundary words in the Shift condition than the No Shift condition.

An analysis of the ARC scores revealed, at best, a marginally significant effect of Event Shifts, *F*(1, 79) = 2.87, *p* = .09, *η*_*p*_^*2*^ = .04, with ARC scores actually being lower in the Shift condition (*M* = .48; *SE* = .04), than in the No Shift condition (*M* = .57; *SE* = .04). Thus, there was no immediate evidence of event structure improving the chunking of information. Overall, the pattern of data for the immediate test is at odds with those of Pettijohn et al. (2016). There was no overall benefit of a Shift condition, and there was not more clustering. Finally, while that study found no difference between memory for Boundary and Nonboundary items, a difference was present here.

#### Changes over time

**Fig. 6:**
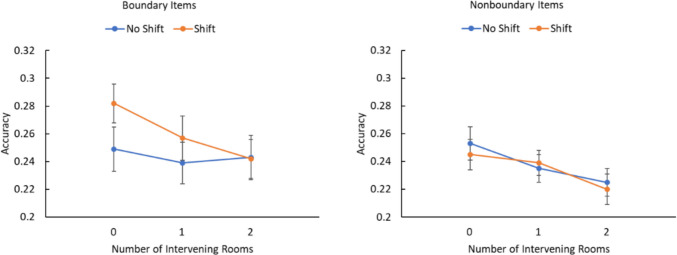
Accuracy scores for the No Shift and Shift conditions as a function of the Number of Intervening Rooms between learning and test. (Color figure online)

Now we move from the attempted replication to look at how the patterns of data changed over time (Fig. 6). First, the mean recall accuracy for the word lists was .24 (*SE* = .01). The accuracy data were submitted to a 3 (Intervening Rooms: 0, 1, or 2) × 2 (Event Shifts: No Shift versus Shift) × 2 (Boundary: Nonboundary versus Boundary) mixed ANOVA, with the first variable being between-subjects and the other two within. This analysis revealed no main effect of Number of Intervening Rooms, *F*(2, 237) = 1.35, *p* = .21, *η*_*p*_^*2*^ = .01, or Event Shifts, *F*(1, 237) = 1.81, *p* = .01, *η*_*p*_^*2*^ = .01, but there was a main effect of Boundary, *F*(1, 237) = 6.17, *p* = .01, *η*_*p*_^*2*^ = .03, with people having better memory for the Boundary (*M* = .25; *SE* = .01) than the Nonboundary words (*M* = .23; *SE* = .01). There was also an Event Shifts x Boundary Interaction, *F*(1, 237) = 3.89, *p* = .05, *η*_*p*_^*2*^ = .02. The other interactions were not significant, all *p v*alues > .30.

To address the interaction, we analyzed the data from the Nonboundary and Boundary conditions separately. For the Nonboundary items, there were no main effects of the Number of Intervening Rooms, *F*(2, 237) = 1.84, *p* = .16, *η*_*p*_^*2*^ = .02, or Event Shifts, *F* < 1, nor an interaction, *F* < 1. However, for the Boundary items, there was a marginally significant main effect of Event Shifts, *F*(1, 237) = 3.16, *p* = .08, *η*_*p*_^*2*^ = .01, with people having better memory in the Shift (*M* = .26; *SE* = .01) than the No Shift condition (*M* = .24; *SE* = .01). There was no main effect of Number of Intervening Rooms, *F* < 1, nor an interaction, *F*(2, 237) = 1.08, *p* = .34, *η*_*p*_^*2*^ = .01. Overall, while there are some hints at differences and changes over time with this data, much of it is quite weak, depending on marginally significant effects and nominal data trends.

**Fig. 7:**
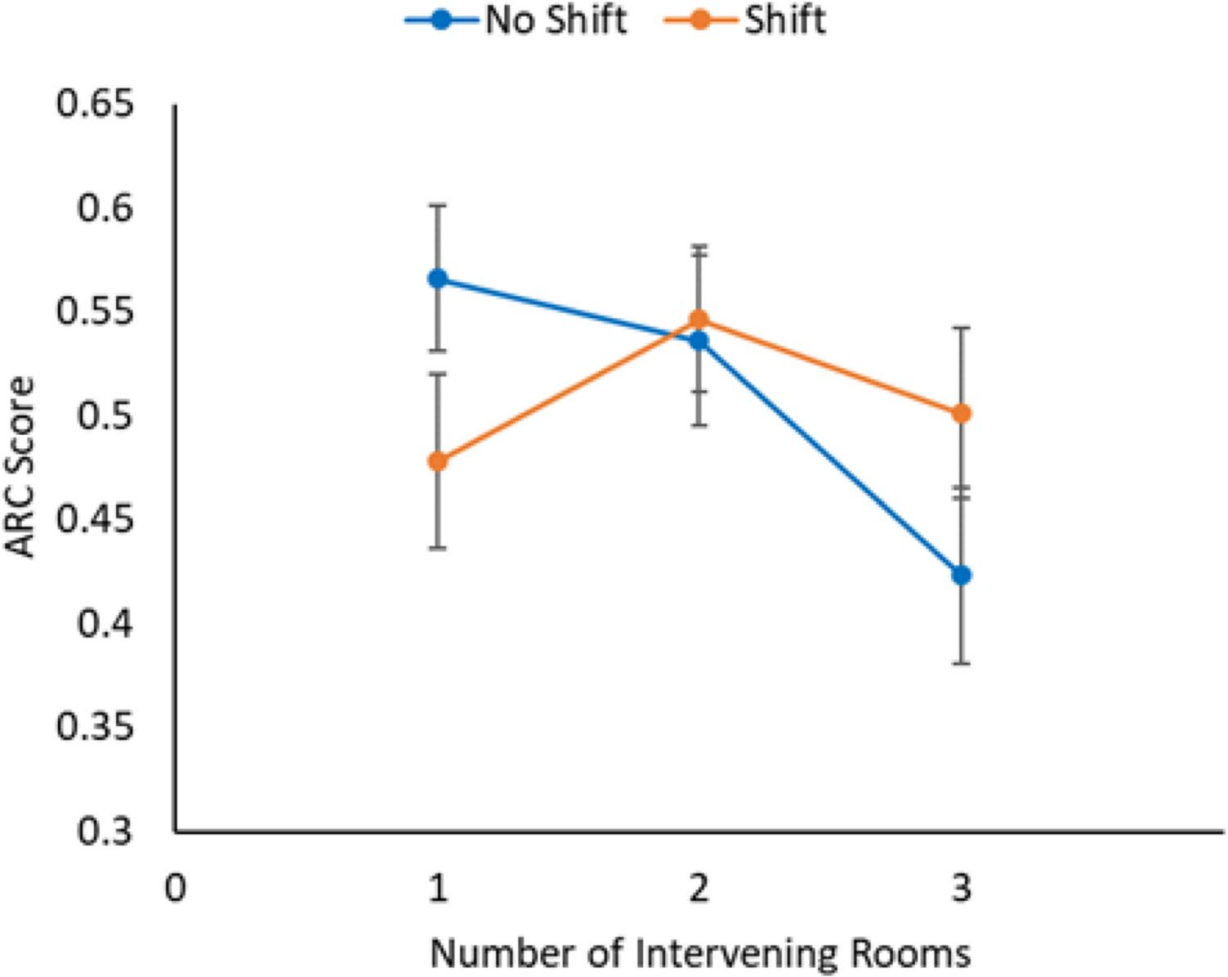
ARC scores for the No Shift and Shift conditions as a function of the Number of Intervening Rooms between learning and test. (Color figure online)

We also analyzed the ARC score data. This analysis revealed no main effect of the Number of Intervening Rooms, *F*(2, 237) = 1.90, *p* = .15, *η*_*p*_^*2*^ = .02, nor Event Shifts, *F* < 1. The interaction was marginally significant, *F*(2, 237) = 2.62, *p* = .075, *η*_*p*_^*2*^ = .02. As can be seen in Fig. 7, for the No Shift condition, there was a main effect of the Number of Intervening Rooms, *F*(2, 237) = 3.63, *p* = .03, *η*_*p*_^*2*^ = .03, with ARC scores decreasing with more rooms. However, there was no such difference for the Shift condition, *F* < 1. The impact of different learning locations had a decreasing impact on memory when the words were learned in a single room (i.e., the two tables within a larger room) but not when they were learned in two different rooms. This is perhaps because in a single room, the information is integrated into one larger event, while two different rooms created a distinctive quality. So, overall, the data again are inconsistent with those reported by Pettijohn et al. (2016). Moreover, the present effects are quite weak.

#### Serial position

**Fig. 8:**
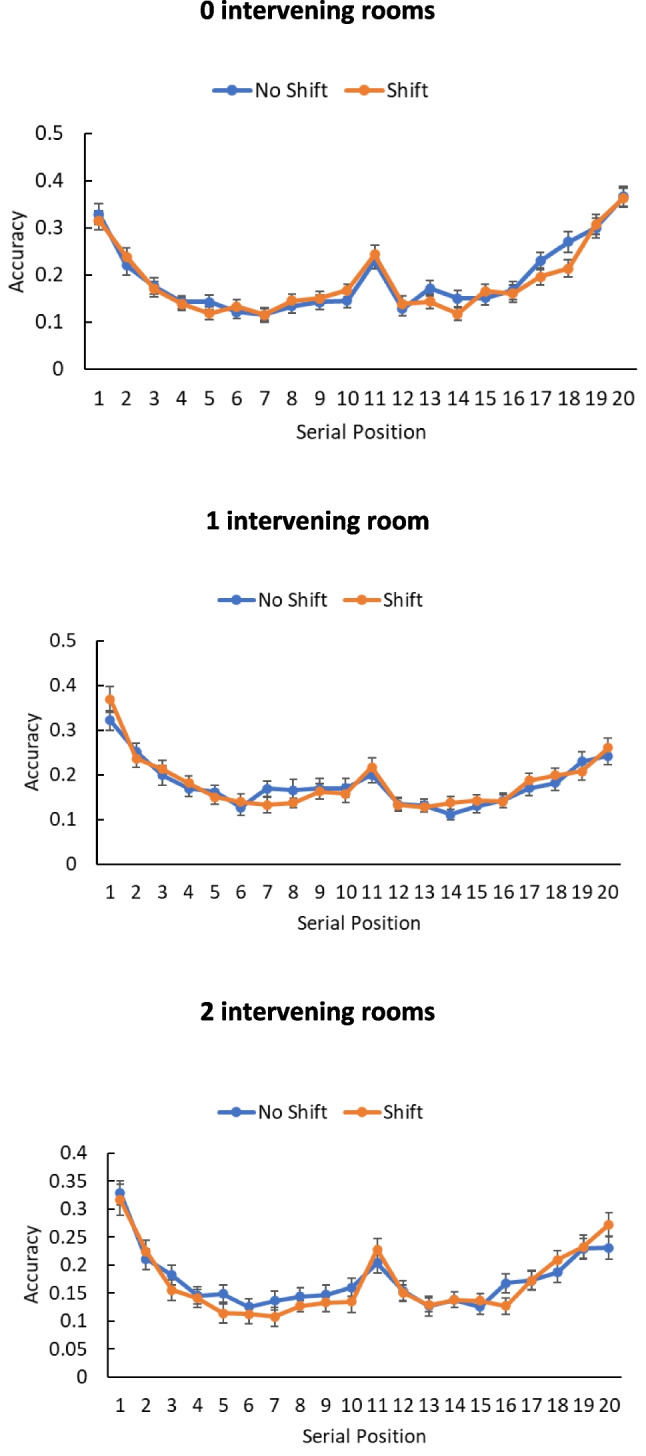
Serial position curves for the No Shift and Shift conditions for different numbers of intervening rooms for Experiment 2

We again looked at serial position memory, and whether this was affected by the list halves being experienced as part of a single event or two. The serial position data are shown in Fig. 8. Again, although there is an increase in memory between the list halves, this was unaffected by whether the halves were read in the same or different rooms. These data were submitted to a 3 (Intervening Rooms: 0, 1, or 2) × 2 (Event Shifts: No Shift versus Shift) × 20 (Serial Position) mixed ANOVA. Again, there was a main effect of Serial Position, *F*(19, 4503) = 64.34, *p* < .001, *η*_*p*_^*2*^ = .21, which is not surprising given the presence of serial position curves. There were no main effects of number of Intervening Rooms, *F*(2, 237) = 1.42, *p* = .24, *η*_*p*_^*2*^ = .01, or Event Shifts, *F*(1, 237) = 0.35, *p* = .55, *η*_*p*_^*2*^ = .001, but there was an Intervening Rooms × Serial Position interaction, *F*(38, 4503) = 2.99, *p* < .001, *η*_*p*_^*2*^ = .03, with a smaller recency effect for end list items. This is a classic effect when there is longer delay between the end of a set and recall (Glanzer & Cunitz, 1966). None of the other interactions were significant, all *p* values ≥ 36.

## General discussion

The results of the current study show that there were few differences between No Shift and Shift conditions. Although Pettijohn et al. (2016) reported an overall memory accuracy benefit in the Shift condition, this was not seen here. Such a benefit only emerged in Experiment 2 when there was more power, and only when there were no intervening rooms between the end of the study list and the testing room, and only for the Boundary items. Additionally, for Experiment 1, there was a very small, at best, marginally significant effect in the opposite direction. Thus, overall, in terms of accuracy, there is no clear benefit from dividing list learning into multiple events, and the effects that are present are weak and inconsistent.

In terms of the difference in memory for boundary and nonboundary items, there are several studies with more complex materials that show a memory benefit for information occurring near an event boundary (e.g., Gold et al., 2017; Sonne et al., 2017; Swallow et al., 2009). A similar benefit for words coming just before or just after a shift from one location to another was observed for Experiment 2. This boundary effect was weakened when additional rooms to navigate were added, likely due to an increased separation between learning and the memory test. Thus, the effect appears to be easily disrupted by having multiple rooms between the end of list presentation and memory testing. It is not clear if the superior memory for items occurring near an event boundary would persist in other studies given that these studies only assessed this at a single time-point. Notably, there was no memory benefit for boundary items in Pettijohn et al.’s (2016) study.

As for the ARC score measure of clustering, Pettijohn et al. (2016) observed no difference. For both of our experiments, we also failed to find a reliable effect. There was a marginal difference in Experiment 2, which suggests an unreliable effect. Moreover, the direction was the opposite of what would be predicted. This runs counter to the idea that segregating the word list into separate events could aid memory by increasing clustering. It should also be noted that this organizational pattern did not survive the transition across subsequent events, and shows a trend towards reversal, particularly for the No Shift condition. Thus, there may be organizational benefit that is obscured initially. However, even here, the evidence is weak.

Another dimension that was assessed here was the influence of interposing a number of additional events between the end of the list learning and recall memory testing. Prior work has shown that imposing rooms between information influences the accessibility of that information, with the critical factor being the number of rooms, not their size (Rinck et al., 1997). While not significant, there was a general trend for memories to be worse with more intervening rooms. What is particularly notable is that the differences between No Shift and Shift conditions that were present when there was no intervening room went away when intervening rooms were present. This may be because as people move further from the original learning locations, the separate, less distinct subevents across the initial room(s) may be blended into a larger mental model of a single event of encountering the word list. Thus, the segregation of the material into list halves decreased. Another possibility, and consistent with the pattern of data overall, is that each instance of an intervening room involved walking through more and more doorways. Each of these instances would have resulted in more forgetting (cf. Radvansky et al., 2011), leading to changes in performance.

Overall, despite the inconsistency in the data, there does appear to be an influence of event structure on word list memory. However, it is subtle and not always apparent in a given measure. This was the case even though, compared with Pettijohn et al. (2016) we had greater statistical power, with more participants and more observations per person.

The absence of strong effects of event structure on word list memory may be due to our use of virtual rather than real environments. People may not have felt as immersed in the various rooms, and thus, the impact of shifts from one room to another may not have had as great of an impact. Moreover, most if not all of the people viewed the environments on smaller screens than is typically done in some other studies, and this can reduce the impact of event shifts (Radvansky et al., 2011). It should be noted that the current study tested people over the internet, whereas the study by Pettijohn et al. (2016) was done in the lab. It may be that people were not as engaged with the event structures in the current study to affect word list memory. This idea is further supported by studies of improved memory when people learn materials in different physical spatial contexts (Bilodeau & Schlosberg, 1951; Greenspoon & Ranyard, 1957; Jensen et al., 1971; Smith, 1982, 1984; Smith & Rothkopf, 1984; Smith et al., 1978; Strand, 1970).

Another explanation may have to do with the nature of the materials used in this study. In particular, we assessed memory for lists of words, which is a fairly impoverished source of information. In another study with a very similar design, people read narratives as they navigated an online virtual environment. They were then tested for their memories of the narratives later (Crockett et al., 2025). This study found that there were clear influences of event structure on memory. When the reading of a given text was divided into two rooms, compared with all of it being read in one room, memory was worse at the surface form (verbatim memory) and textbase (propositional memory) levels, but actually better at the event model level (understanding). Thus, effects of event structure, in the way it was manipulated here, can be observed on memory, but with more complex materials.

Finally, another explanation for these findings may be related to the fact that information was divided into only two events. Work by Logie and Donaldson (2021) revealed that people remembered lists of words better when they were segregated into 10 different events, and that ARC score measures of clustering can also reflect this^2^. Thus, we can conclude that dividing information into two events results, at best, in a mild improvement of world list memory, with most of the benefit observed for Boundary items. Dividing information into a larger number of events can show a larger benefit, but seems to limit its practicality.

Overall, the current work further explored the idea that event structure can influence later memory. The results revealed some differences in memory for the organization of word lists, but these differences were limited, and did not persist when there were other rooms between learning and testing. Because the effects of event structure have been observed on memory in other studies, the current study may not have done so because of the impoverished nature of the materials, the reduced immersion in the environments, and the number of events the information was spread across. Thus, this work provides some limiting conditions on when such event cognition effects may be observed.

## Data Availability

The datasets generated during the current study are available in the OSF repository: https://osf.io/gpckf/

## Footnotes

1 ARC scores are calculated by taking the number of within category repetitions across the number of categories in a person’s recall report relative to the number of category repetitions that would occur by chance.

2 It should be noted that people also viewed the items longer in the `Logie and Donaldson (2021) study: 3 s versus our 1.

## Code availability

The software code used to collect data for this study is available upon request.
